# A Simplified Kinetic Model for the Enantioselective Hydrogenation of 1-Phenyl-1,2-Propanedione over Ir/TiO_2_ in the Presence of a Chiral Additive

**DOI:** 10.1021/acs.iecr.1c04375

**Published:** 2022-04-25

**Authors:** Ignacio Melián-Cabrera, Teresita Marzialetti, M. Fernanda Neira D’Angelo, Cristian H. Campos, Patricio Reyes

**Affiliations:** † Applied Photochemistry and Materials for Energy Group, University of La Laguna, Avda. Astrofísico Francisco Sánchez, s/n, P.O. Box 456, 38200 San Cristóbal de La Laguna, S/C de Tenerife, Spain; ‡ Chemical Engineering Department, School of Engineering, Universidad de Concepción, Edmundo Larenas 219, Concepción 4070409, Chile; § Laboratory of Chemical Reactor Engineering, Department of Chemical Engineering and Chemistry, Eindhoven University of Technology, P.O. Box 513, 5600 MB, Eindhoven, The Netherlands; ∥ Departamento de Físico-Química, Facultad de Ciencias Químicas, Universidad de Concepción, Edmundo Larenas 129, 4070371, Concepción, Chile; ⊥ Sustainable Process Engineering, Department of Chemical Engineering and Chemistry, Eindhoven University of Technology, P.O. Box 513, 5600 MB, Eindhoven, The Netherlands

## Abstract

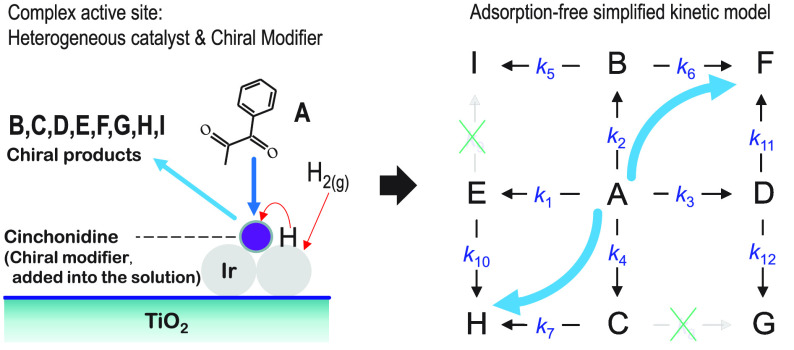

This communication proposes a preliminary simplified kinetic model for the hydrogenation of 1-phenyl-1,2-propanedione that can render up to eight compounds, involving regioselectivity and enantioselectivity. The catalytic system comprises two functionalities; the heterogeneous catalyst (Ir/TiO_2_) plays the role for the hydrogenation, whereas the adsorption/binding to the active site is played by a chiral molecule (cinchonidine), added to the reaction mixture. The reaction occurs at room temperature and total pressure of 40 bar. The product distribution shows competitive parallel and series pathways with up to 12 possible reactions. Despite the complexity of both reaction and catalyst system, a simplified kinetic model was able to predict the concentrations profiles. The model assumes the reactions to be apparent first order in the concentrations of reactant and intermediate products, while the kinetic constants include all other effects (partial pressure of hydrogen, solvent and catalyst effects, and the concentration of the chiral additive). The concentration profiles were well-modeled with low residual values. The errors in the kinetic constants (*k*-values) were small for all relevant parameters of the main reaction pathways. Two *k*-values are nil, which is the lower bound imposed in the model, suggesting that these reaction pathways are likely negligible. The positive outcome from this simplified model suggests that the process can be formally treated as a first-order irreversible homogeneous catalyzed reaction, despite a heterogeneous catalyst was employed (with a modifier). Despite the promising results, the model must be extended for a more general applicability, or conditions where it is applicable.

In heterogeneous catalysis, selectivity is a crucial property.^1−3^ Among the most challenging topics in selective heterogeneous catalysis is the concept of enantioselective synthesis (ES). In ES, a prochiral compound is transformed to a chiral product in the presence of an optically active aid, such as solvents, additives, reactants, or catalysts.^4,5^ ES has been applied to synthesize molecules of interest for various sectors such as pharmaceuticals, vitamins, agrochemicals, flavors, and fragrances, as well as functional materials.^6,7^

The hydrogenation of prochiral diones has drawn significant attention in the past using heterogeneous catalysts.^8−12^ This reaction involves concepts of regioselectivity and enantioselectivity, depending on which carbonyl is hydrogenated (regio), and the produced *R* or *S* isomers (enantio). A chiral auxiliary molecule denoted as modifier enables this reaction. The modifier leads the reactant, or intermediates, to be adsorbed on the active site in a certain way. In addition to that, supported noble metals are used to activate the hydrogen molecule. The conformation of the modifier is the key to directing the reaction selectivity. For instance, cinchonidine (CD), a typically employed modifier, exhibits two *closed* and four *open* conformers; in an *open* conformation, the lone pair of the quinuclidine nitrogen points away from the quinoline ring, whereas the *closed* points toward the quinoline ring.^10,13,14^ It is widely accepted that one of the open conformers involves the enantio-differentiating ability.^10^

A reaction of applied interest is the hydrogenation of 1-phenyl-1,2-propanedione (**A** in Figure 1) using various supported noble metals with CD as the modifier.^8−12^ The modifier can be added to the solution or grafted onto the inorganic support. The active site model consists of two domains; the hydrogenation function is given by a heterogeneous catalyst (Ir/TiO_2_ in this case), whereas the adsorption/binding site is a chiral molecule (modifier), cinchonidine in this case. The active site models are schematically shown in Figure S1 in the Supporting Information. Figure S1(1) represents a catalytic system with the modifier added into the solution (but likely physically adsorbed onto the heterogeneous catalyst surface), while Figure S1(2) shows the modifier grafted onto the support. The former case was employed in this work. The reaction proceeds by hydrogenating the carbonyl next to the phenyl ring, leading to products **B** and **C**, or by hydrogenating the additional carbonyl leading to **D** and **E**. The reaction can continue by hydrogenating the second carbonyl, leading to four diols: **F**, **G**, **H**, and **I**. The hydrogenation of the aromatic ring does not occur. There is hardly any literature on this reaction showing that it is purely irreversible. Hence, the reaction network can include irreversible and reversible steps. Because of the shape of the concentration profiles (explained in more detail later), we considered irreversible steps in Figure 1, and the model later on. Among all these products, compound **B** ((*R*)-1-hydroxy-1-phenylpropanone, also known as *L*-PAC) is a crucial intermediate in the pharmaceutical sector.^15,16^ It is produced via a microbial biotransformation process using different species of yeasts from benzaldehyde. Efforts have been devoted to replacing this process by a heterogeneously catalyzed approach. Therefore, it is essential to understand the kinetic behavior to optimize this compound’s yield. In this regard, a kinetic analysis is a powerful mathematical tool^17,18^ to quantify the roles of metal, support, solvent and, in this case, the modifier concentration on the yield of the desired product. In other words, having kinetic parameters is more useful than using yields when analyzing trends among catalysts. A kinetic expression also allows one to determine the suitable reactor design (high or low concentration of reactants and temperature control) and reactor size.^19^

**Figure 1:**
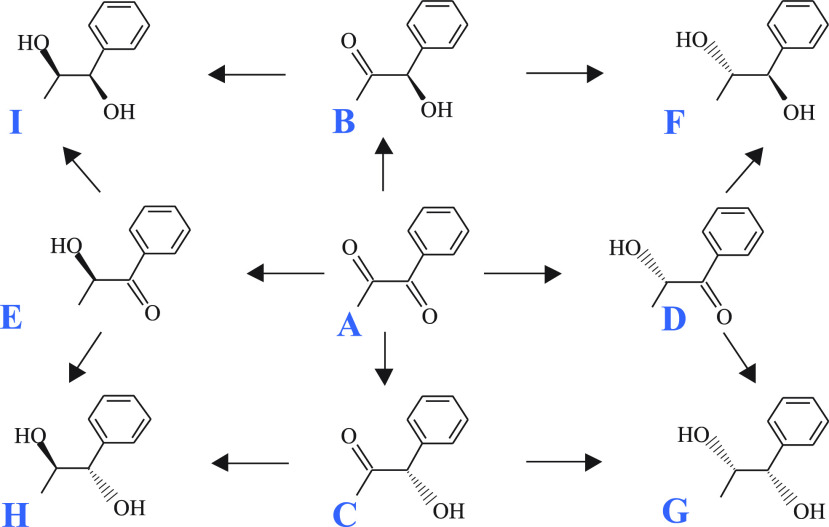
Reaction pathways for the hydrogenation of 1-phenyl-1,2-propanedione in the presence of cinchonidine (not shown for clarity) into hydroxyketones (**B**–**E**) and diols (**F**–**I**): (**A**) 1-phenyl-1,2-propanedione; (**B**) (*R*)-1-hydroxy-1-phenylpropanone; (**C**) (*S*)-1-hydroxy-1-phenylpropanone; (**D**) (*S*)-2-hydroxy-1-phenylpropanone; (**E**) (*R*)-2-hydroxy-1-phenylpropanone; (**F**) (1*R*,2*S*)-1-phenyl-1,2-propanediol; (**G**) (1*S*,2*S*)-1-phenyl-1,2-propanediol; (**H**) (1*S*,2*R*)-1-phenyl-1,2-propanediol; and (**I**) (1*R*,2*R*)-1-phenyl-1,2-propanediol.

Toukoniitty et al. proposed a 16-parameter kinetic model to fit the experimental data for the hydrogenation of **A**.^9^ They assumed two adsorption sites for the reactant, two adsorption sites for the modifier, and two additional sites for the forming substrate–modifier complexes. The model assumed isothermal operation and adsorption equilibria for all the adsorbed species. It predicted the concentration–time profile for all the components, although three parameters displayed a high relative error (>100%).

The inspection of our experimental results for an Ir/TiO_2_ catalyst (Figure 2) shows that the concentration–time profiles of the hydroxyketones (**B**, **C**, **D**, **E**) follow an expected trend for intermediates, with a maximum for each one (Figure 2(2)). At the studied reaction-time range (0–360 min), the concentration of end products **F** and **H** (the more-produced diols) increases continuously without any apparent equilibria boundaries (Figure 2(3)). The concentration of diols **G** and **I** is small to witness, with accuracy, the presence of equilibria or not. The upper reaction time limit at 360 min was applied in the model because the product of interest is compound **B**, which is an intermediate whose concentration’s maximum was already achieved at ca. 180 min and then decreases.

**Figure 2:**
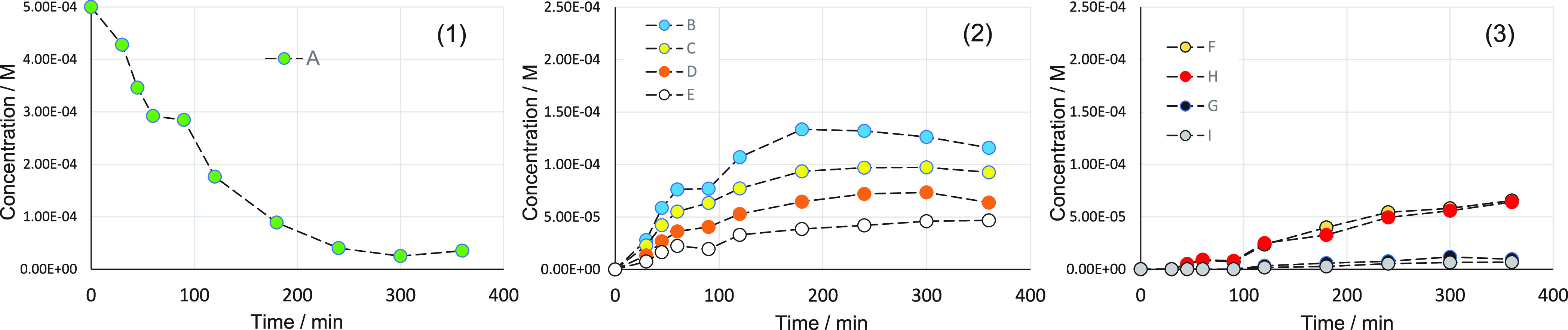
Experimentally observed concentrations for: (**1**) reactant (**A**), (**2**) hydroxyketones (**B**, **C**, **D**, **E**), and (**3**) diols (**F**, **G**, **H**, **I**). Compound codes (**A**–**I**) are given in Figure 1. Hydrogenation reaction of 1-phenyl-1,2-propanedione (**A**) catalyzed by a Ir/TiO_2_ catalyst at room temperature and 40 bar, in the presence of cinchonidine as chiral modifier. Dashed lines are meant to connect the experimental data points.

Based on these observations, we explored whether a simplified power-law irreversible kinetic model can predict the concentration profiles. The model is sketched in Figure 3 (left) and considers the following assumptions: (1) the effect of the hydrogen partial pressure is included in the rate constants; (2) the rate constants also consider the effect of catalyst, solvent, modifier, and temperature, altogether; (3) the surface reactions are the rate-determining step, and they are irreversible first-order reactions (i.e., dehydrogenations do not occur), and (4) adsorption/desorption effects are negligible, and the rate constants include any adsorption contribution. The mathematical formalism for such a simplified model can be found in Figure 3 (right). The parameter estimation was performed for 10 experiments, corresponding to each reaction time. The model predictions were obtained by solving nine ordinary differential equations for all the components, using the diagonal covariance Bayesian solver in Athena Visual Studio, with an initial guess for all the *k*-values of 0.01 min^–1^, and a lower bound of zero.

**Figure 3:**
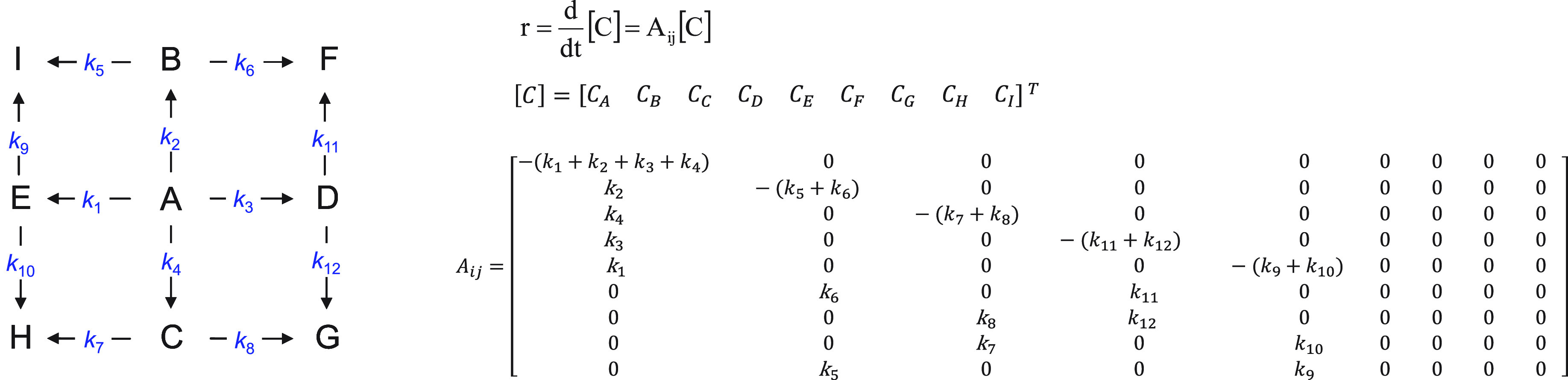
Proposed simplified kinetic model for the hydrogenation of 1-phenyl-1,2-propanedione in the presence of cinchonidine over a Ir/TiO_2_ catalyst. The kinetic model equations have been represented as a matrix formulation, where the superscript “*T*” means transpose. Compound codes (**A**–**I**) are given in Figure 1.

Table 1 lists the obtained parameters, including the error and the overall residuals. The error is given as the 95% marginal highest probability density (HPD) intervals.

**Table 1:** Kinetic Constants Derived from the Model^a^

*k* (min^–1^)	value	95% marginal intervals^b^
*k*_1_	0.821 × 10^–3^	±0.099 × 10^–3^
*k*_2_	3.175 × 10^–3^	±0.248 × 10^–3^
*k*_3_	1.472 × 10^–3^	±0.124 × 10^–3^
*k*_4_	2.501 × 10^–3^	±0.221 × 10^–3^
*k*_5_	0.199 × 10^–3^	±0.019 × 10^–3^
*k*_6_	1.776 × 10^–3^	±0.325 × 10^–3^
*k*_7_	2.285 × 10^–3^	±0.484 × 10^–3^
*k*_8_	0^c^	
*k*_9_	0^c^	
*k*_10_	0.241 × 10^–3^	±0.780 × 10^–3^
*k*_11_	0.359 × 10^–3^	±0.516 × 10^–3^
*k*_12_	0.617 × 10^–3^	±0.084 × 10^–3^

a Sum of squares of residuals: 7.64161 × 10^–9^.

b Error given as the 95% marginal highest probability density (HPD) intervals; i.e., 95% probability that the true estimate would lie within the lower and upper limits of the interval.

c Lower bound imposed in the model. The solver fits it to zero when any parameter becomes negative during the computation.

The *k*-values for the main reactions (i.e., reactions yielding **B**, **C**, **D**, and **E**) are in agreement with the experimental results, in the sense that the higher *k*-value, the higher the optimal (i.e., maximum) concentration (Figure 2(2)), i.e., *k*_2_ > *k*_4_ > *k*_3_ > *k*_1_. The error for these parameters is smaller than the values themselves, which indicates the goodness of the fit. As for the secondary products (**F**, **G**, **H**, **I**), some *k*-values are nil (*k*_8_ and *k*_9_), i.e., the lower bound imposed in the model, suggesting that these reaction pathways are likely negligible. When we run the model without any constraints, these parameters provided small but negative values, which have no physical meaning. That is the reason why we added a lower bound at zero for all parameters. Two *k*-values have a relatively high error (*k*_10_ and *k*_11_) and can be tentatively associated with the high dispersion of experimental data for **F** and **H** (see concentration profiles for **F** and **H** in Figure 4, where the residuals are high compared to the absolute values). However, these high errors do not affect the interpretation of the concentration profiles for **F** and **H** (more-produced diols); the absolute values of *k*_2_ and *k*_6_ (to explain a high **F**) are higher than *k*_3_ and *k*_11_, whereas the absolute values of *k*_4_ and *k*_7_ (to explain a high **H**) are higher than *k*_1_ and *k*_10_.

**Figure 4:**
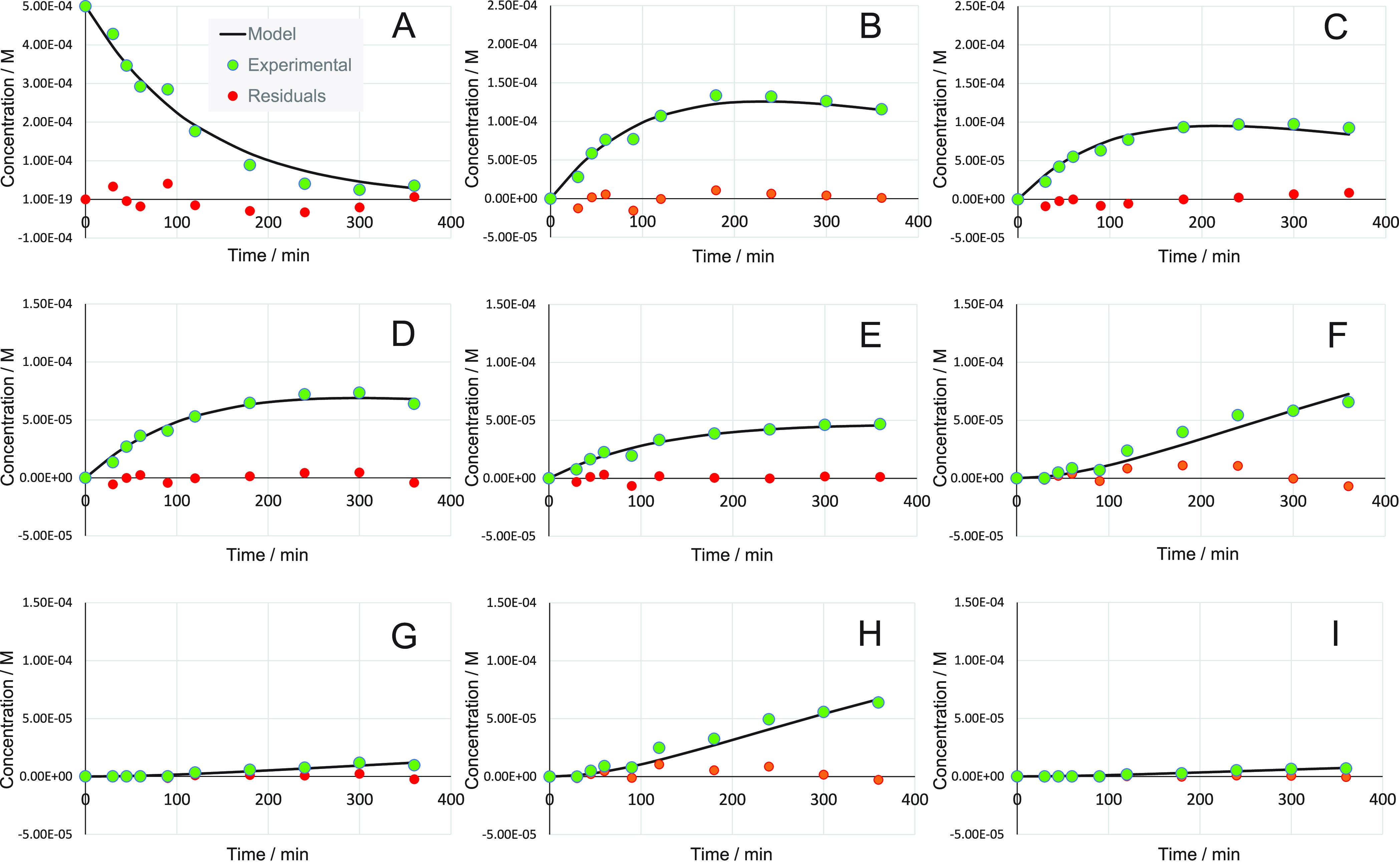
Model results for the hydrogenation reaction of 1-phenyl-1,2-propanedione catalyzed by a Ir/TiO_2_ catalyst at room temperature and 40 bar, in the presence of cinchonidine as a chiral modifier. Compound codes (**A**–**I**) are given in Figure 1.

The correlation matrix for the estimated parameters, given in Table S1 in the Supporting Information, shows no severe interdependence among the fitted parameters (i.e., all correlation factors are lower than 0.9), which highlight the robustness of this simplified kinetic model. Nevertheless, the pairs *k*_10_↔*k*_1_ and *k*_10_↔*k*_7_ describing the parallel pathways toward **H** show a relatively higher interdependence (i.e., correlation factors of 0.86 and 0.81, respectively).

Additional experiments by (co)feeding **E** or **C** could lead to further improvements in the model, but these were not commercially available.

Considering the obtained *k*-values, the predicted concentrations versus time for each component were plotted and compared to the experimental values in Figure 4, including the residuals. The model nicely fitted the experimental data. Yet, the concentration–time profiles of **F** and **H** show some discrepancies between the predicted and experimental data, likely due to the high errors in *k*_10_ and *k*_11_.

As a summary of the model results, based on the obtained *k*-values, Figure 5 provides a graphical outline showing the negligible routes, i.e., **E**–**I** and **C**–**G**, and preferential pathways in this catalyzed process, i.e., **A**–**B**–**F** and **A**–**C**–**H**. The other routes can be considered intermediate (**A**–**D**–**F, A**–**D**–**G**, **A**–**B**–**I**, and **A**–**E**–**H)**.

**Figure 5:**
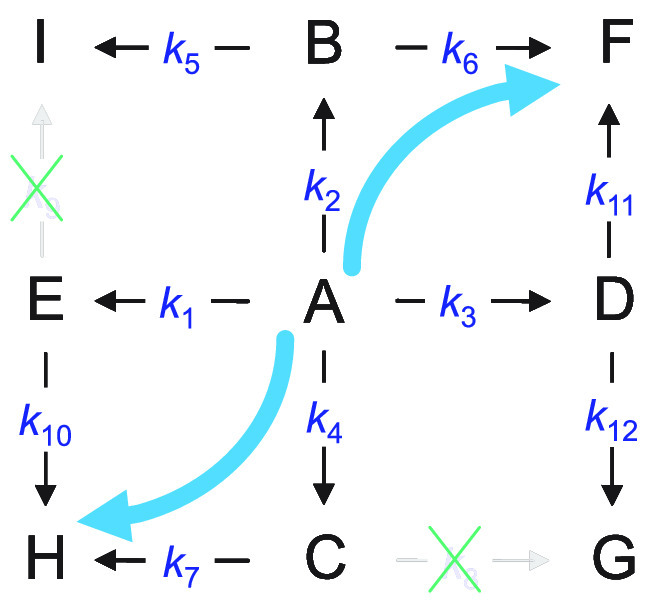
Summary of the results for the simplified kinetic model, showing the negligible (crossed out) and preferential routes (thick lines), for the hydrogenation of 1-phenyl-1,2-propanedione in the presence of cinchonidine over a Ir/TiO_2_ catalyst. Compound codes (**A**–**I**) are given in Figure 1.

In comparison to a previous model proposed for this reaction comprising adsorption effects for the chiral modifier, reactant, and modifier-reactant complexes,^9^ this simplified model explains well the concentration profiles. This suggests that the process can be considered from a mathematical standpoint as a first-order irreversible homogeneously catalyzed reaction, although a heterogeneous catalyst (with a modifier) is employed. This is fundamentally interesting and a novel insight for this reaction. In practical terms, the result is attractive when considering that a simpler model is easier to implement in, e.g., a process simulator for plant design and optimization purposes, although it may not show the intrinsic phenomena.

Surface coverage, adsorption, and desorption have been widely used to interpret the kinetics of heterogeneous catalysis. Here, we report a case in which these phenomena can be ignored. There are several ways to explain this effect where dilution of the substrate appears to be the key factor. This can be expanded in a future work.

In conclusion, a simplified kinetic model considering irreversible first-order reactions predicted the concentration–time profiles of all pathways involved in the hydrogenation of 1-phenyl-1,2-propanedione, catalyzed by an Ir/TiO_2_ catalyst and cinchonidine as chiral modifier. There are two aspects to be improved: reducing the scattering of the experimental data to decrease the relative errors in the model parameters, and extending the model to more catalysts and broader reaction conditions to validate its general applicability, or conditions where it is applicable.

## Experimental Methods

The experimental methods are described in the Supporting Information.

## Modeling Methodology

Parameter estimation within the kinetic model was performed using the Athena Visual Workbench, using ordinary differential equations and the diagonal covariance Bayesian solver method, with an initial guess for all the *k*-values of 0.01 min^–1^, and a lower bound of zero. The latter condition means that the solver fits the parameter to zero when it becomes negative during the computation.

## Supporting Information Available

Experimental methods; active site models; normalized parameter covariance matrix; typical gas chromatogram showing the position of the reaction compounds (PDF)

## Dedication

In memory of Prof. José Luis García Fierro.
