# Supplementary material for: A Simplified Kinetic Model for the Enantioselective Hydrogenation of 1-Phenyl-1,2-Propanedione over Ir/TiO2 in the Presence of a Chiral Additive
Source: Ind Eng Chem Res. 2022 Apr 25;61(17):6052–6. doi: 10.1021/acs.iecr.1c04375 (PMC10394662; doi:10.1021/acs.iecr.1c04375)
Supplement: Supplementary file 1 — ie1c04375_si_001.pdf [file ie1c04375_si_001.pdf]

# Supporting Information

# A Simplified Kinetic Model for the Enantioselective Hydrogenation of 1-Phenyl-1,2-Propanedione over $Ir/TiO_2$ in the Presence of a Chiral Additive

Ignacio Melián-Cabrera,[a,*] Teresita Marzialetti,[b] M. Fernanda Neira D'Angelo,[c,d] Cristian H. Campos[e] and Patricio Reyes[e,*]

a. Applied Photochemistry and Materials for Energy Group, University of La Laguna, Avda. Astrofísico Francisco Sánchez, s/n, P.O. Box 456, 38200 San Cristóbal de La Laguna, S/C de Tenerife, Spain.

b. Chemical Engineering Department, School of Engineering, Universidad de Concepción, Edmundo Larenas 219, 4070409, Concepción, Chile.

c. Laboratory of Chemical Reactor Engineering, Department of Chemical Engineering and Chemistry, Eindhoven University of Technology, P.O. Box 513, 5600 MB, Eindhoven, The Netherlands.

d. Sustainable Process Engineering, Department of Chemical Engineering and Chemistry, Eindhoven University of Technology, P.O. Box 513, 5600 MB, Eindhoven, The Netherlands.

e. Departamento de Físico-Química, Facultad de Ciencias Químicas, Universidad de Concepción, Edmundo Larenas 129, 4070371, Concepción, Chile.

Corresponding authors:

ignacio.melian.cabrera@ull.edu.es

preyes@udec.cl

# Additional information

## Experimental methods

***Chemicals*****.** All the chemicals employed in this study were supplied by Sigma-Aldrich at the highest purity available, except for the solvent, cyclohexane, supplied by Merk (GR for analysis). The $TiO_2$ support was kindly provided by Degussa (P-25).

***Catalyst*****.** The Ir/$TiO_2$ catalyst preparation followed the method reported by Marzialetti et al.[1] following the low-temperature approach meaning that the catalyst was reduced in $H_2$ at 573 K.

***Reaction*****.** The hydrogenation of 1-phenyl-1,2-propanedione (**A**) was carried out in a type-316 stainless steel Parr reactor at room temperature (i.e. ~298 K) and a constant hydrogen pressure of 40 bar. The initial concentration of **A** was $5\times10^{-4}$ M. Cinchonidine at a concentration of $3.4\times10^{-4}$ M was used as a chiral modifier. Around hundred mg of powdered catalyst was reduced *ex situ* in a $H_2$ flow at 573 K and maintained at this temperature for 2 h. The catalyst was transferred into the solvent (cyclohexane) in a fume hood, and the slurry was transferred into the reactor vessel. Subsequently, the modifier, substrate and the remaining solvent (50 mL in total) were added into the vessel. The reactor was closed, purged with a flow of ultra-pure $N_2$ to remove atmospheric air and stabilized at room temperature. The $N_2$ atmosphere was swept out with a pure $H_2$ flow and, after a stabilization time, the reactor was pressurized at 40 bar $H_2$, and the stirring speed was set at 900 rpm, which determined the start of the reaction. Samples were drawn out from the reactor and were analyzed offline by gas chromatography using a 30-m β-Dex 225 chiral column (Supelco). A typical gas chromatogram can be found in Figure S2 (below), which indicates the retention times for each compound. The reader may refer to Marzialetti et al.[1] for more details about the analytical procedure or other publications from Prof. Reyes' group.

(1) Marzialetti, T.; Fierro. J. L. G.; Reyes, P. Iridium-supported catalyst for enantioselective hydrogenation of 1-phenyl-1,2-propanedione: The effects of the addition of promoter and the modifier concentration. *Catal. Today* **2005**, *107–108*, 235–243.

# Additional Figures and Tables

**Figure S1.** Graphical sketches illustrating the models for the active sites…………………………Page 3

**Table S1**. Normalized parameter covariance matrix…………………………………………………………Page 4

**Figure S2**. A typical gas chromatogram showing the position of the reaction compounds….Page 5

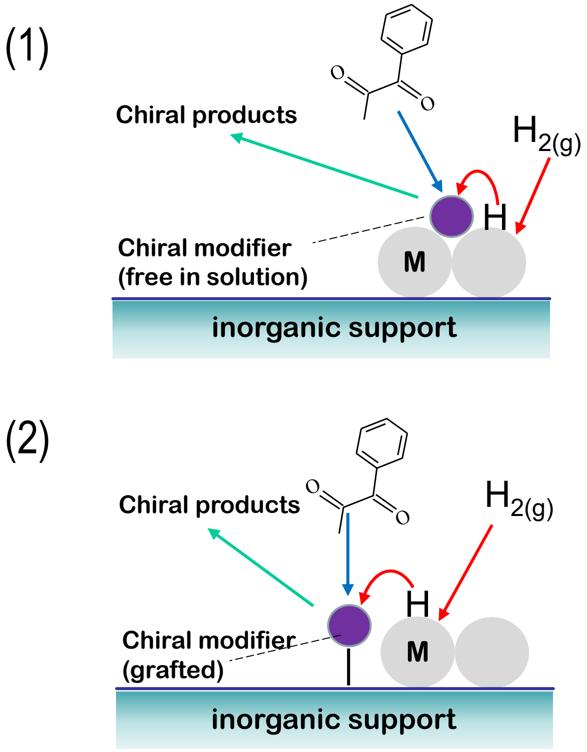


**Figure S1.** Graphical sketches illustrating the models for the active sites: (**1**) the chiral modifier is added free in the solution (it is here assumed to be adsorbed on the surface of the metal particles); (**2**) the chiral modifier is grafted on the support's surface. For simplicity, the chiral modifier is shown here as spherical domain but its conformation is crucial.

**Table S1**. Normalized parameter covariance matrix.

| | $k_1$ | $k_2$ | $k_3$ | $k_4$ | $k_5$ | $k_6$ | $k_7$ | $k_8$ | $k_9$ | $k_{10}$ | $k_{11}$ | $k_{12}$ |
|---|---|---|---|---|---|---|---|---|---|---|---|---|
| $k_1$ | 1.000 | | | | | | | | | | | |
| $k_2$ | -0.007 | 1.000 | | | | | | | | | | |
| $k_3$ | -0.002 | -0.106 | 1.000 | | | | | | | | | |
| $k_4$ | -0.420 | 0.536 | 0.237 | 1.000 | | | | | | | | |
| $k_5$ | 0.008 | -0.380 | -0.010 | -0.151 | 1.000 | | | | | | | |
| $k_6$ | -0.001 | 0.468 | -0.590 | 0.102 | 0.021 | 1.000 | | | | | | |
| $k_7$ | -0.665 | 0.279 | 0.123 | 0.712 | -0.080 | 0.053 | 1.000 | | | | | |
| $k_8$ | 0 | 0 | 0 | 0 | 0 | 0 | 0 | 0 | | | | |
| $k_9$ | 0 | 0 | 0 | 0 | 0 | 0 | 0 | 0 | 0 | | | |
| $k_{10}$ | 0.863 | -0.262 | -0.113 | -0.683 | 0.089 | -0.049 | -0.810 | 0 | 0 | 1.000 | | |
| $k_{11}$ | -0.003 | -0.542 | 0.765 | -0.141 | 0.120 | -0.763 | -0.072 | 0 | 0 | 0.057 | 1.000 | |
| $k_{12}$ | 0.000 | 0.080 | -0.202 | -0.018 | -0.009 | 0.181 | -0.009 | 0 | 0 | 0.007 | -0.235 | 1.000 |

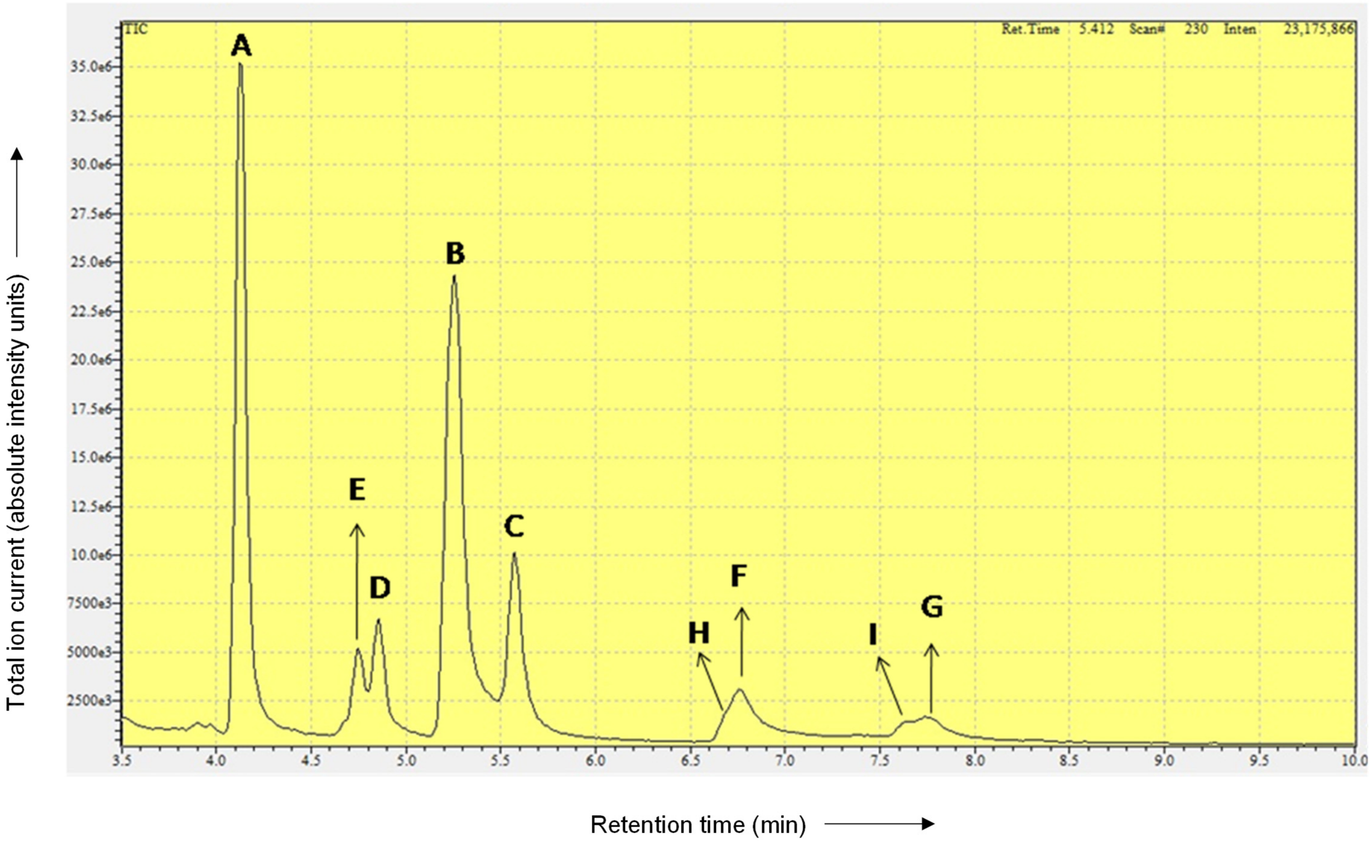


**Figure S2**. A typical gas chromatogram showing the position of the reaction compounds: (**A**) 1-phenyl-1,2-propanedione; (**B**) (R)-1-hydroxy-1-phenylpropanone; (**C**) (S)-1-hydroxy-1-phenylpropanone; (**D**) (S)-2-hydroxy-1-phenylpropanone; (**E**) (R)-2-hydroxy-1-phenylpropanone; (**F**) (1R,2S)-1-phenyl-1,2-propanediol; (**G**) (1S,2S)-1-phenyl-1,2-propanediol; (**H**) (1S,2R)-1-phenyl-1,2-propanediol and (**I**) (1R,2R)-1-phenyl-1,2-propanediol.
